# Coupled Resonance Enhanced Modulation for a Graphene-Loaded Metamaterial Absorber

**DOI:** 10.1186/s11671-019-2852-y

**Published:** 2019-01-22

**Authors:** Dong Xiao, Qiang Liu, Lei Lei, Yiling Sun, Zhengbiao Ouyang, Keyu Tao

**Affiliations:** THz Technical Research Center of Shenzhen University, Shenzhen Key Laboratory of Micro-Nano Photonic Information Technology, Key Laboratory of Optoelectronic Devices and Systems of Ministry of Education and Guangdong Province, College of Electronic Science and Technology, Shenzhen University, Shenzhen, 518060 China

**Keywords:** Metamaterials, Graphene, Broadband modulation, Coupled-resonance, Mid-infrared absorber

## Abstract

A graphene-loaded metamaterial absorber is investigated in the mid-infrared region. The light-graphene interaction is greatly enhanced by virtue of the coupled resonance through a cross-shaped slot. The absorption peaks show a significant blueshift with increasing Fermi level, enabling a wide range of tunability for the absorber. A simple circuit model well explains and predicts this modulation behavior. Our proposal may find applications in a variety of areas such as switching, sensing, modulating, and biochemical detecting.

**Electronic supplementary material:**

The online version of this article (10.1186/s11671-019-2852-y) contains supplementary material, which is available to authorized users.

## Background

Plasmonic metamaterial (PM) absorbers work with metallic nanostructures at deep subwavelength scale. Perfect absorptions can be achieved and tailored at particular wavelengths, leading to a variety of applications including light emitter/detector, sensor, photothermal therapy, optical-mechanical interaction, and hyperspectral imaging [1–7]. PM absorbers also provide a promising platform for designing novel functional devices with tunable properties. By introducing components such as liquid crystals, semiconductors, or phase-change materials, the optical response can be modulated electrically, optically, or thermally [8–13], which enables new types of modulators, switches, and multispectral detectors.

Most recently, graphene has received considerable attention because of its high-speed modulation capability and tunability as a plasmonic material [14–20]. Specifically, the graphene conductivity depends on the Fermi level (*E*_F_) which can be continuously tuned through bias voltage within several nanoseconds, enabling a high modulation rate in the near infrared and mid-infrared regions [17, 19–24]. However, as the single graphene layer is only atomically thick, the interaction between the incident light and the plasmonic resonance is quite weak. And this interaction becomes even weaker in the mid-infrared area due to the Pauli blocking of interband transitions [22]. As a result, the wavelength tuning range as well as the modulation depth is quite limited. The wavelength shift is generally less than 10% of the resonance wavelength [21, 22, 25–28], which is still a challenge for practical applications in optical communications and wideband spectral detections. Thus, in order to achieve efficient electro-optical modulation, the graphene-light interaction needs to be greatly strengthened. Some progresses have been made in previous studies. Based on the designs of complex nanostructures such as nano-antennas and split ring resonators [19, 21, 22, 25, 27, 28], the enhancement of graphene-light interaction has been theoretically and experimentally demonstrated. Yet, these designs are usually complicated or polarization-dependent, the range of working frequency is relatively small and the tunability is still limited.

In this work, we have proposed a graphene-loaded absorber with modulation range from 9 to 14 μm, which is of great interest for applications such as biochemical sensing and thermal imaging [5, 29–31]. The coupled-resonances inside the cross-shaped slot offer four orders of enhancement for the electric field, strongly intensifying the graphene-light interaction and resulting in a shift of up to 25% in the central wavelength. In addition, we propose a simple LC circuit model which well explains and predicts the graphene-induced modulation controlled by the voltage and geometric parameters. Such a large range of tunability would be promising in many applications.

## Methods

**Fig. 1:**
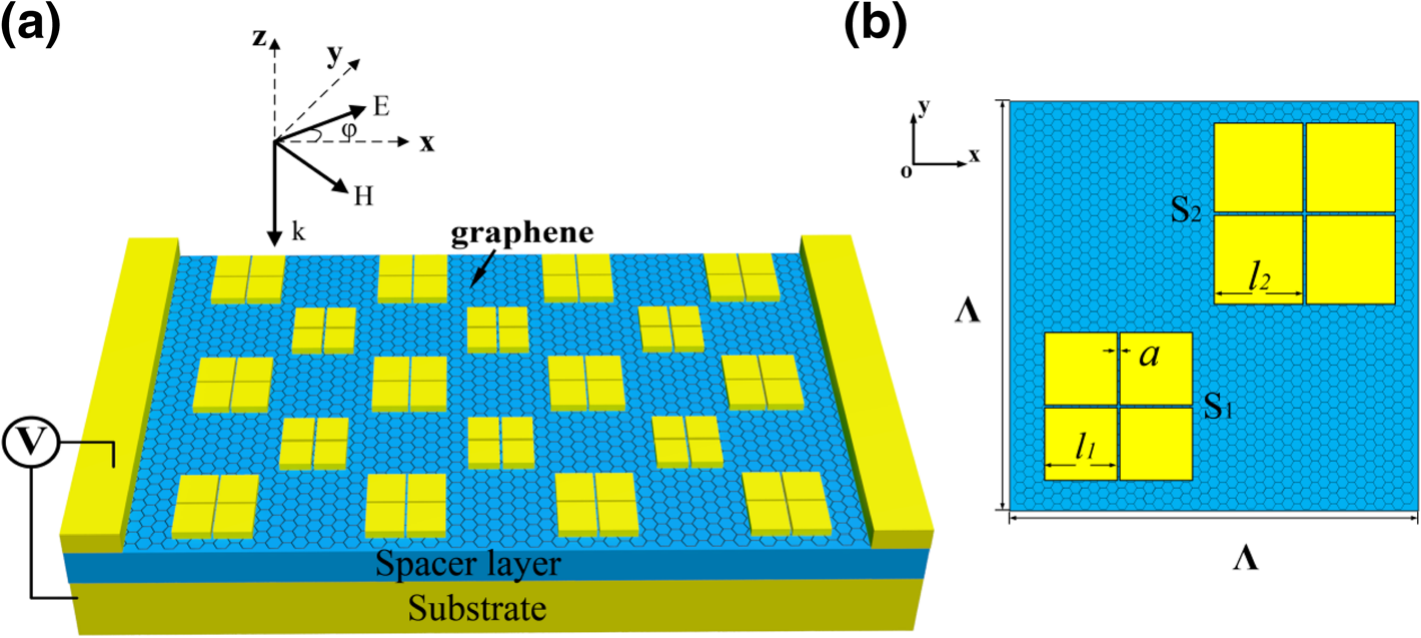
**a** Schematic diagram of the proposed graphene-loaded metamaterial. The cross-shaped slot in each subunit enables a great enhancement of graphene-light interaction without polarization dependence. **b** Top view of the structure in one period. Two subunits are arranged diagonally with different patch sizes

As shown in Fig. 1a, patterned metallic patches are arranged with a period of Λ = 8 μm on the metal substrate separated by a dielectric spacer. A single layer of graphene sandwiches between the patches and the spacer. The substrate is very thick and acts as a reflection mirror. The thickness of the spacer layer is *t*_d_ = 520 nm and that of the metallic patches is *t*_m_ = 100 nm. Figure 1b shows the top view of one unit cell. Two subunits are arranged in a diagonal symmetry in order to support the polarization independence. A cross-shaped slot is etched on each square patch, dividing it into four small identicals. The sizes of the small identicals in *S*_1_ and *S*_2_ are *l*_1_ = 1.5 μm and *l*_2_ = 1.7 μm, respectively. The slot width for both subunits is *a* = 20 nm. In our study, the metallic material is chosen as gold (Au), whose optical property is described by the Drude model of $\varepsilon \left(\omega \right)=1-{\omega}_p^2/\left(\omega \left(\omega +\tau \right)\right)$ with *ω*_*p*_ = 1.369 × 10^16^ Hz and *τ* = 1.224 × 10^14^ Hz [32]. The dielectric spacer is composed of zinc sulfide (ZnS), whose optical index is *n* = 2.2 with negligible loss in the mid-infrared region [33].

The finite-difference time-domain (FDTD; Lumerical FDTD Solutions) method is employed to calculate reflectance spectra and electromagnetic field distribution. The simulations are carried out with periodic boundary conditions in the *x* and *y* directions and perfect matched layer conditions in the *z* directions. The single graphene layer is modeled as a two-dimensional structure by the surface conductivity approach [34]. The surface conductivity of the graphene layer *σ*_g_, including the interband term *σ*_inter_ and the intraband term *σ*_intra_, can be calculated by the Kubo formula [35].

1 $$ {\displaystyle \begin{array}{l}{\sigma}_{\mathrm{g}}\left(\omega, {E}_{\mathrm{F}},\Gamma, T\right)={\sigma}_{\mathrm{intra}}+{\sigma}_{\mathrm{inter}}\\ {}=\frac{-{ie}^2}{\pi {\mathrm{\hslash}}^2\left(\omega +i2\Gamma \right)}\underset{0}{\overset{\infty }{\int }}\xi \left(\frac{\partial {f}_d\left(\xi \right)}{\partial \xi }-\frac{\partial {f}_d\left(-\xi \right)}{\partial \xi}\right) d\xi +\frac{ie^2\left(\omega +i2\Gamma \right)}{\pi {\mathrm{\hslash}}^2}\underset{0}{\overset{\infty }{\int }}\xi \left(\frac{f_d\left(-\xi \right)-{f}_d\left(\xi \right)}{{\left(\omega +i2\Gamma \right)}^2-4{\left(\xi /\mathrm{\hslash}\right)}^2}\right) d\xi \end{array}} $$

where *e* and *ξ* are the charge and energy of the electron, ℏ is the reduced plank’s constant, *ω* is the angular frequency, ${f}_d\equiv 1/\left({e}^{\left(\xi -{E}_F\right)/{k}_BT}+1\right)$ refers to the Fermi-Dirac distribution, *T* is the absolute temperature, *Γ* is the scattering rate, *k*_*B*_ is the Boltzmann constant, and *E*_F_ is the Fermi level. In our calculation, *T* = 300 K, and *Γ* = 10 meV [28]. The mesh size near the graphene layer is 0.25 nm, and 2.5 nm in the slots. The effective permittivity of graphene can then be expressed as

2 $$ {\varepsilon}_{\mathrm{g}}=1+\mathrm{i}{\sigma}_{\mathrm{g}}/\left({\varepsilon}_0\omega {t}_{\mathrm{g}}\right) $$

where *ε*_0_ is the permittivity of vacuum, and *t*_g_ is the thickness of graphene layer. Equations (1) and (2) demonstrate that the optical constants of graphene change with *E*_F_. This change leads to tunability of the absorption frequency, whose range can be greatly enlarged by the coupled resonance in the nanostructures, substantially lowering the applied voltage in devices.

## Results and Discussion

**Fig. 2:**
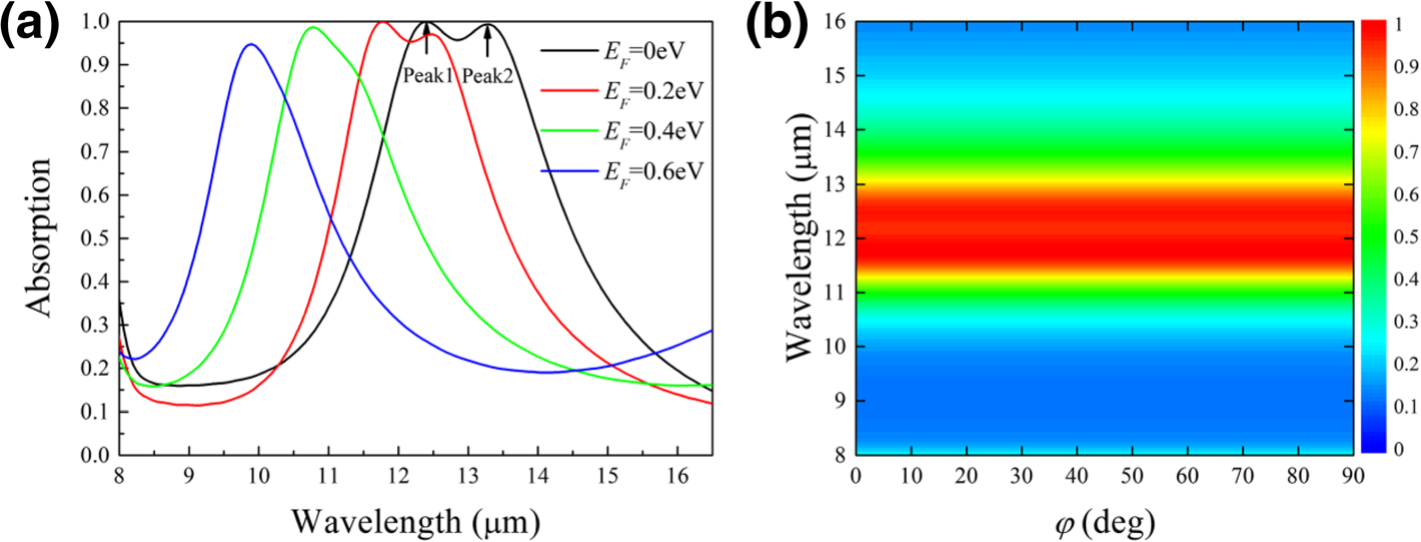
Absorption spectra at the normal incidence with different *E*_*F*_ at *φ* = 0, showing large blueshift of the peaks with increasing *E*_*F*_ (**a**), and with different *φ* at *E*_*F*_ = 0.2eV, demonstrating a polarization independence (**b**). The polarization angle *φ* is defined as in Fig. 1a

Figure 2a shows the absorption spectra for *x*-polarized wave (*φ* = 0) at the normal incidence. When the Fermi level is *E*_F_ = 0eV, two absorption peaks are observed at the wavelength *λ* = 12.4 μm and 13.3 μm, respectively. The incident light ranging from 12.1 to 13.5 μm is almost absorbed by the nanostructure. As *E*_F_ increases, the resonance moves toward shorter wavelength. At *E*_F_ = 0.2 eV, the absorption peaks shift to 11.8 μm and 12.46 μm, indicating respectively a relative shift of 4.8% and 6%. Meanwhile, the absorbance of peak 2 declines, which is attributed to the impedance mismatch between the metamaterial and air at a higher *E*_F_ [28]. Here, it is interesting that peak 2 blueshifts faster than peak 1 as the Fermi level keeps increasing. This observed behavior will be explained later by a circuit model.

The modulation can be quantified by a parameter *M* = Δ*λ*/*λ*_0_, where *λ*_0_ is the resonance wavelength at *E*_F_ = 0 eV and Δ*λ* is the wavelength shift due to the change of *E*_F_. Figure 2a shows *M*_1_ = 20.1% and *M*_2_ = 25.5% for peak 1 and peak 2, respectively, when *E*_F_ reaches 0.6 eV. The modulation range of resonances is much broader compared with previous works [19, 21, 22, 25–28]. Such a large modulation at a low *E*_F_ is highly desirable for many applications. Separate calculations show that the absorption peaks blueshift with decreasing thickness of the spacer (Additional file 1). Thus, we can optimize the thickness to set a suitable start point of modulation. In addition, the optical response of the proposed metamaterial is polarization-independent as shown in Fig. 2b. The absorption spectrum keeps unchanged when the polarization angle *φ* varies from 0 to 90°, owing to the symmetry of the design.

**Fig. 3:**
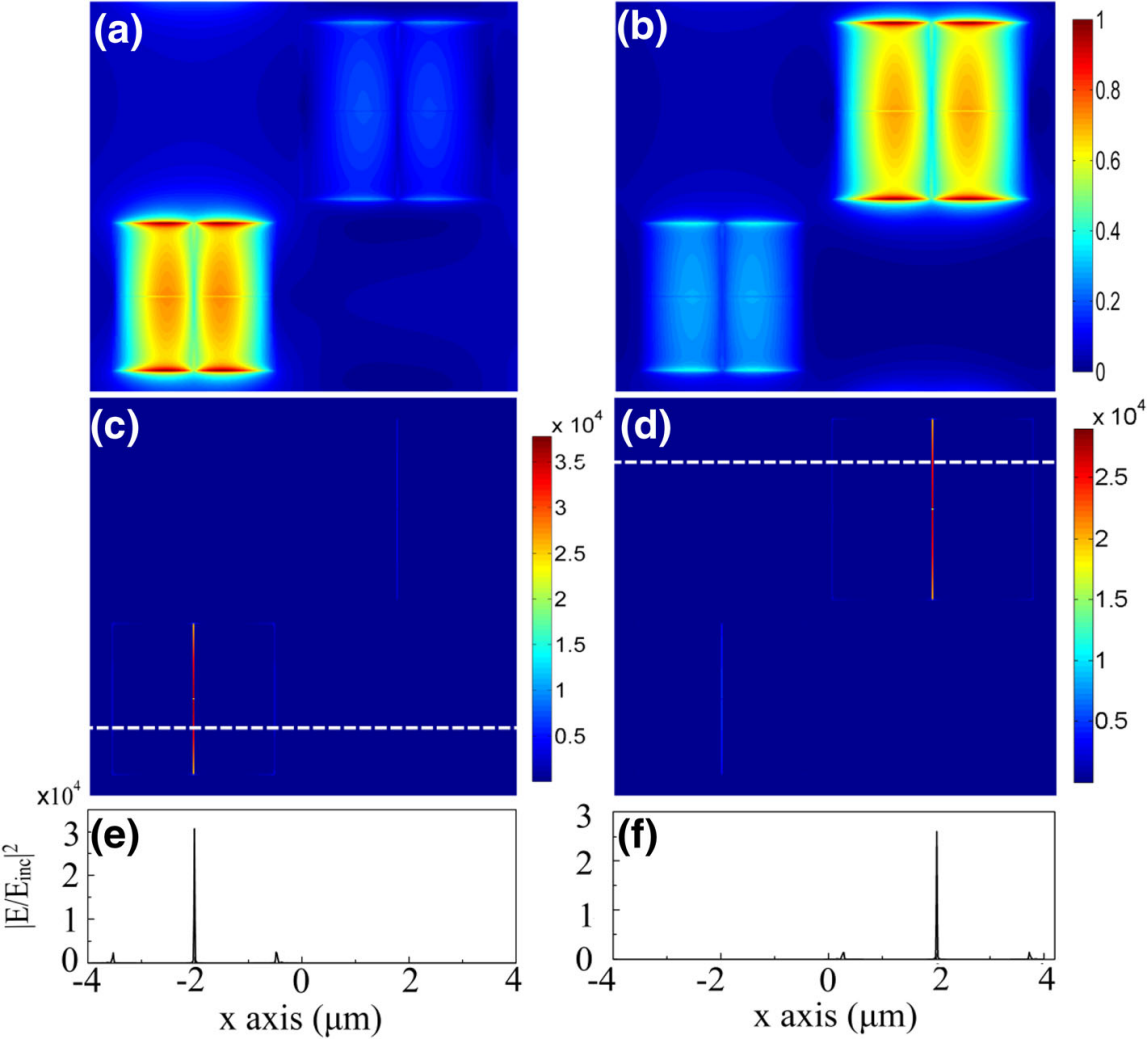
Field distributions in the graphene layer at *E*_*F*_ = 0.2 eV for the x-polarized light at the normal incidence, showing great enhancement in the slot caused by the coupled-resonances. **a, b** Normalized magnetic field |H|^2^ at *λ*_1_ = 11.8 μm (**a**) and *λ*_2_ = 12.46 μm (**b**); **c**, **d** The corresponding field distribution of |E/E_inc_|^2^; **e**, **f** |E/E_inc_|^2^ along the white dash-line shown in **c** and **d**, respectively. A sharp contrast is seen between the intensity inside the slot and that at the patch edges, giving hints for much wider tuning range than that in previous works

The mechanism of perfect absorptions is clearly illustrated by the field distributions at the resonances. Because of the well-known metal-insulator-metal (MIM) structure [3, 32, 36–38] shown in Fig. 1, localized SPPs are stimulated to form compact magnetic resonances in each patch. Figure 3a and b demonstrate the normalized magnetic field |H|^2^ in the graphene layer for *E*_F_ = 0.2 eV at the resonance wavelengths of *λ*_1_ = 11.8 μm and *λ*_2_ = 12.46 μm, respectively. Since the SPPs are strongly localized, two subunits can work independently. However, due to the narrow width of the splitting slot inside each subunit, the resonances of the four identicals are actually coupled to each other. And this coupling tremendously increases the electric field inside the slot, as shown in Fig. 3c and d. Only the *E* fields in the *y*-direction slot are obvious here because the incident light is in the *x* polarization. The intensity of the *E* field enhanced by the resonance coupling is four orders of magnitude larger than that of the incident light *E*_inc_. In contrast, the most intensified fields used for modulation in previous work are at the patch edges. Figure 3e and f show the sharp comparison of the enhancements between the slots and edges along the white line in Fig. 3c and d, respectively.

Such field distributions well explain the reason why the modulation is so great in our proposal. Based on a perturbation theory, the graphene-induced shifting of resonance can be evaluated as Δ*ω* =  − *iσ*_*g*_∫_*S*_|E_s_|^2^*dS*/*W*_0_ [22]. Here, |E_s_|^2^is the intensity of the electric field in the graphene layer, *W*_*0*_ is the stored energy, and *S* denotes the area covered by the graphene. The spectral shift of the resonance (Re(Δ*ω*)) is decided by the imaginary part of *σ*_g_, which is much greater than its real part in the mid-infrared region [22, 28]. As clearly shown in the Fig. 3c–f, the enhancement of electric field inside the narrow slot is more than 10 times of that at the edges. As a result, the integral value is mainly contributed by the greatly enhanced *E* field in the patch slots, leading to a much bigger shift of the peaks than in previous cases which only possess the enhanced *E* fields at the metallic edges [21, 22, 25, 27, 28].

**Fig. 4:**
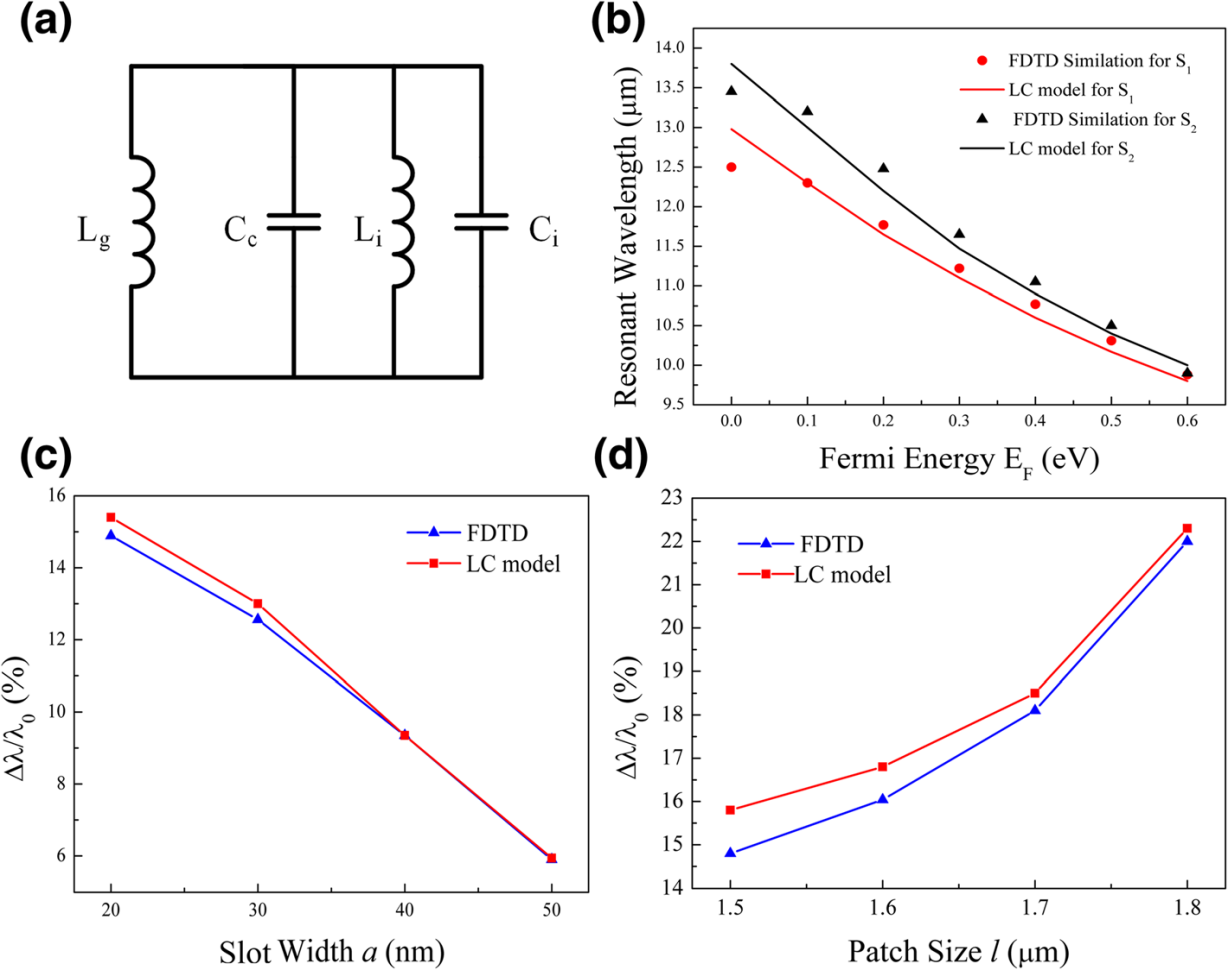
**a** An LC circuit model includes contributions from separate patches (*L*_*i*_and *C*_*i*_), slot (*C*_*c*_), and graphene (*L*_*g*_). **b** Resonances calculated by the LC model compared with FDTD simulations. **c**, **d** Resonance shift for a single patch at *E*_*F*_ = 0.4 eV with changing geometrical parameter of **c** slot width (*l* = 1.5 μm) and **d** patch size (*a* = 20 nm)

According to the field distributions and above discussions, an LC circuit model is proposed to study the tuning behavior. As shown in Fig. 4a, *L*_*i*_ and *C*_*i*_ (*i* = 1, 2) are, respectively, the inductance and capacitance for the patch *S*_*i*_ in Fig. 1b. When the slot width *a* is very big and there is no graphene layer, we can ignore the effects induced by the slots and graphene. Then, *L*_*i*_ and *C*_*i*_ can be decided by separate calculations through fitting with the resonant wavelength obtained in absorption spectra [37, 39, 40]. The results are *L*_1_ = 0.07 pH and *C*_1_ = 350 aF for subunit *S*_1_, while *L*_2_ = 0.075 pH and *C*_2_ = 380 aF for subunit *S*_2_. The slot-induced coupling effect inside each subunit can be described by a shunt capacitance *C*_*c*_, which is found to decrease with the increasing slot width *a*. In our cases, *C*_*c*_ is 290 aF for *a* = 20 nm, and becomes 200 aF, 180 aF, and 135 aF with every increasing 10 nm of *a*. The resonance wavelength is obtained by letting the impedance of the circuit to be zero, i.e., ${\lambda}_i^0=2\pi {c}_0\sqrt{L_i{\mathrm{C}}_i^0}$. Here, *c*_0_ is the light speed in vacuum, “*i*” refers to subunit *S*_*i*_, and ${C}_i^0={C}_i+{C}_c$.

The two-dimensional graphene layer basically acts as an inductor. As shown in Fig. 3, the main contribution of the graphene layer comes from the slot position where the electric field is intensified. Since the slot width is much smaller than the operating wavelength and the wavelength of graphene plasmon, the quasi-static approximation is valid. The voltage *V* and the current *I* across the slot can be evaluated by *V* = *aE* and*I* = 2*l*_i_*t*_g_(*σ*_g_ − *iωε*_0_)*E*, where *E* is the electric field in the graphene layer. So, we can introduce an inductance *L*_g_ =  − 1/*ω* Im(V/I) [41], which describes the contribution of the graphene layer and is found to be

3 $$ {L}_{\mathrm{g}}=\frac{a}{2{l}_i{\omega}^2{\varepsilon}_0\left|\operatorname{Re}\left({\varepsilon}_{\mathrm{g}}\right)\right|{t}_{\mathrm{g}}}\kern0.5em \left(i=1,2\right) $$

This inductor serves as a parallel element shown in Fig. 4a. As a result, the total inductance of one patch is obtained by $1/{L}_i^{\prime }=1/{L}_i+1/{L}_{\mathrm{g}}$. The final resonance wavelength of each subunit, with the graphene layer, becomes

4 $$ {\lambda}_i^{\prime }=2\pi {c}_0\sqrt{L_i^{\prime }{\mathrm{C}}_i^0}\kern0.5em \left(i=1,2\right) $$

Because each subunit works independently, the total impedance of the metamaterial can be obtained from the parallel connection of the impedances of the two subunits.

This LC model predicts a blueshift of the resonance with increasing *E*_F_. Deduced from Eqs. (1) and (2), we get a larger value of |Re(*ε*_g_)| for the graphene at a higher *E*_F_, which gives a smaller *L*_g_ in Eq. (3). Because of the parallel connection of the inductors, the final inductor ${L}_i^{\prime }$ becomes smaller, leading to a shorter wavelength of resonance in Eq. (4). The calculated result is summarized in Fig. 4b, showing a good agreement with the resonant wavelength obtained by the FDTD simulations. Small deviation is seen because our LC model ignores the contribution of weak fields at the edges of each patch (Fig. 3c–f). The LC model also shows how the geometric parameters influence the blueshift of the resonance. Differentiating Eq. (4), we have $\partial {\lambda}_i^{\prime }/\partial {L}_i^{\prime}\propto 1/\sqrt{L_i^{\prime }}$. It is obvious that a small value of $\sqrt{L_i^{\prime }}$ is favored to increase the sensitivity of this blueshift. Because the inductors are parallelly connected and *L*_*i*_ is fixed, a small value of the total inductance ${L}_i^{\prime }$ means a small value of the graphene inductance *L*_g_. In order to increase the tuning range, the slot width *a* should be small and the patch size *l* be large, according to Eq. (3). Figure 4c shows that the blueshift of resonance at *E*_F_ = 0.4 eV increases from around 6 to 15%, when the slot width inside *S*_1_ decreases from 50 to 20 nm. On the other hand, if we fix the slot width at *a* = 20 nm, the resonance increases from 15 to 22% with patch size changing from 1.5 to 1.8 μm as shown in Fig. 4d. The good agreement with the FDTD simulations demonstrates that such a simple circuit model is an efficient method for studying related metamaterials devices.

## Conclusions

In conclusion, we have designed a polarization-independent, broadband metamaterial absorber with a large range of modulation. For both resonances, the tuning range reach up to 20.1% and 25.5% of the central wavelength when *E*_F_ increases from 0 to 0.6 eV. Such a large modulation comes from the graphene-light interaction tremendously enhanced by the coupled-resonances inside the cross-shaped slot of each metallic patch. This effect is well described by a graphene-introduced inductor in the LC model. Such a simple model predicts the modulation behavior under different geometric parameters, and the results agree well with the FDTD simulations. Our proposal is beneficial to potential applications such as optical communication, sensing, and thermal imaging.

## Additional file

Additional file 1: Supplementary Material. (PDF 308 kb).

## Abbreviations

*E*_*F*_: Fermi level
FDTD: Finite-different time-domain
MIM: Metal-insulator-metal
PM: Plasmonic metamaterial
ZnS: Zinc sulfide
