# Supplementary material for: Coupled Resonance Enhanced Modulation for a Graphene-Loaded Metamaterial Absorber
Source: Nanoscale Res Lett. 2019 Jan 22;14:32. doi: 10.1186/s11671-019-2852-y (PMC6342740; doi:10.1186/s11671-019-2852-y)
Supplement: Supplementary file 1 — Supplementary Material. (PDF 308 kb). [file 11671_2019_2852_MOESM1_ESM.pdf]

Supplementary Material

1. Influence of the thickness of the spacer layer

The parameters used are as follows: the periodicity is Λ=8 μm, the slot width is $a = 20$ nm, the thickness of the metallic patches is $t_m = 100$ nm, the patch sizes are $l_1 = 1.5$ μm and $l_2 = 1.7$ μm, and the Fermi energy is $E_F = 0.2$ eV.

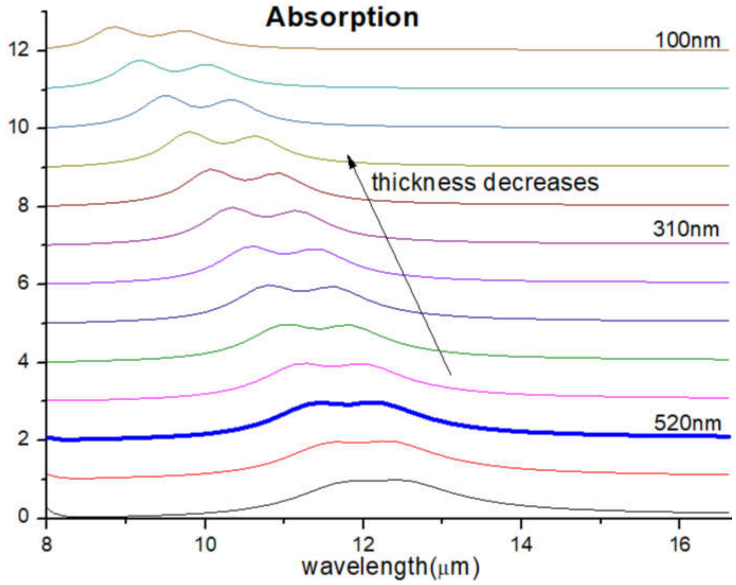

Fig. S1 Absorption spectra on different thickness of the spacer. These curves are separated for clearance by adding an integer to the absorption for each curve.

As shown above, the absorption peaks blueshift with decreasing thickness of the spacer layer. The magnitudes of peaks only decrease obviously when the thickness is below 150 nm, due to the impedance mismatch between the metamaterial with the air. The calculation step is 42 nm in the spacer layer thickness for neighbouring curves. These curves are separated for clearance and simplicity by adding an integer to the absorption for each curve. The blue bold line represents spacer layer thickness of 520 nm used in the main text.

2. Procedure to calculate the inductance and capacitance

The procedure to calculate the inductance and capacitance is as follows.

Step 1. The inductor and capacitor for each patch can be preliminarily determined when there is no graphene layer and when the slot width *a* is big enough. Thus we can ignore the effects induced by graphene and the slots.

Expressions $C_i = \varepsilon_d \varepsilon_0 l_i^2 / t_d$ and $L_i = 0.5\mu_0 l_i t_d$ are generally used to character the capacitor and the inductor, which can be a good start. To reduce the numbers of fitting parameters, we only concern the spectra changing with the patch size and the thickness of spacer. The resonance wavelength is $\lambda = 2\pi c_0 \sqrt{L_i \mathrm{C}_i}$. Meanwhile the resonance wavelength can also be obtained in the absorption spectrum of different l_i and t_d, by FDTD simulations.

We fit LC models with these FDTD data, and found the expressions should be modified as $C_i \propto l_i^{2\alpha} / t_d^{\alpha}$ and $L_i \propto l_i t_d$, concerning the large non-uniform distribution of charge and current (Fig. 3 can be a reference). The exact values are finally optimized after comparing the interaction with the slot-effect C_c.

Step 2. The slot-induced effect C_c, is decided by fitting with the FDTD results at small slot width *a*. The resonance wavelength is now $\lambda' = 2\pi c_0 \sqrt{L_i (C_i + C_c)}$. Then $\lambda'^2 \propto L_i C_i + L_i C_c$ which means $\lambda'^2 \propto \lambda^2 + L_i C_c$. After fitting with groups of data, we can decide C_c, and obtain optimized L_i and C_i based on the results in Step 1.

Step 3. The expression $L_g = \dfrac{a}{2 l_i \omega^2 \varepsilon_0 \left|\mathrm{Re}(\varepsilon_g)\right| t_g}$ is used for the graphene introduced inductor L_g, which serves as a parallel element in the circuit. The resonance wavelength becomes $\lambda' = 2\pi c_0 \sqrt{L_i'(C_i + C_c)}$, where $1/L_i' = 1/L_i + 1/L_g$. The value of L_g can be decided by fitting with the FDTD results with different patch size and slot width under different Fermi energy of the graphene layer.

3. The evolution of absorption spectra

The evolution of absorption spectra are calculated for different Fermi energy in graphene (Figs. S2 and S3) as well as different slot width (Fig. S4) and patch size (Fig. S5).

The parameters used are with Fig. 4 in the main text. Specifically, Fig. S2 and S3 correspond to the FDTD result for S_1 and S_2 in Fig. 4b. Figures S4 and S5 correspond to the FDTD result in Figs. 4c and 4d, respectively.

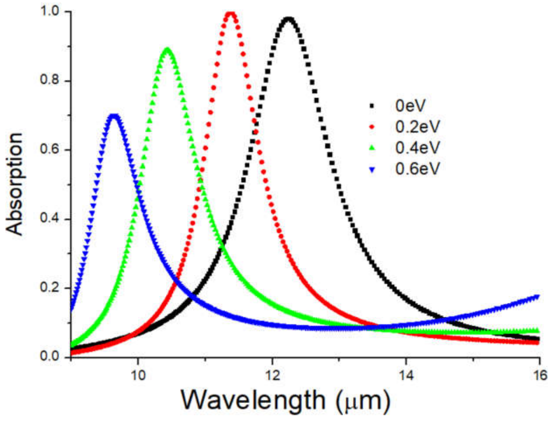

Fig. S2 The evolution of absorption spectra on Fermi energy

in graphene for l = 1.5 μm, a = 20 nm

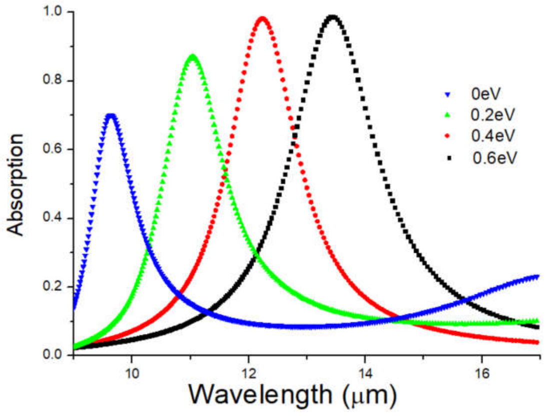

Fig. S3 The evolution of absorption spectra on Fermi energy

in graphene for l = 1.7 μm, a = 20 nm

Figures S2 and S3 show that the absorption peak shifts towards the short wavelength region with increasing Fermi energy of the graphene layer, and the intensity decreases at the same time. However, the maximum intensity at E_F = 0.6eV is still above 70% for both cases.

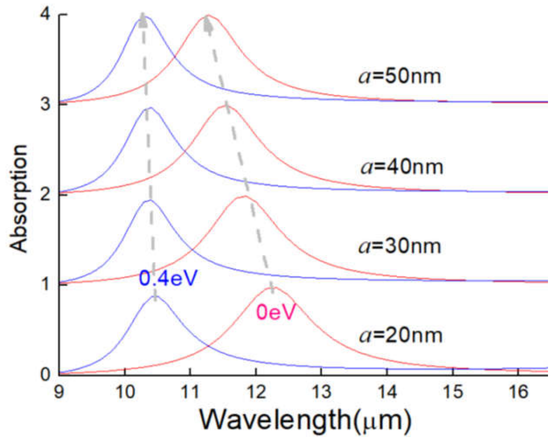

Fig. S4 The evolution of absorption spectra on slot width for l = 1.5 μm.

These curves are separated for clearance by adding an integer to the absorption for each curve.

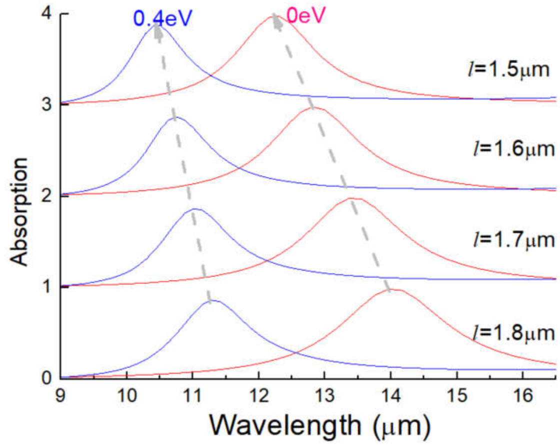

Fig. S5 The evolution of absorption spectra on patch size for a = 20nm.

These curves are separated for clearance by adding an integer to the absorption for each curve.

Figure S4 shows that the absorption peak generally shifts towards the short wavelength region with increasing slot width, meanwhile the intensity does not change

much. Figure S5 shows that the absorption peak shifts towards the short wavelength region with decreasing patch size, but the intensity almost keeps untouched.
